# Evaluation of therapeutic strategies targeting BCAA catabolism using a systems pharmacology model

**DOI:** 10.3389/fphar.2022.993422

**Published:** 2022-11-28

**Authors:** Veronika Voronova, Victor Sokolov, Yannick Morias, Malin Jonsson Boezelman, Maria Wågberg, Marcus Henricsson, Karl Hansson, Alexey Goltsov, Kirill Peskov, Monika Sundqvist

**Affiliations:** ^1^ M&S Decisions LLC, Moscow, Russia; ^2^ STU Sirius, Sochi, Russia; ^3^ Bioscience Cardiovascular, Research and Early Development, Cardiovascular Renal and Metabolism (CVRM), BioPharmaceutical R&D AstraZeneca, Gothenburg, Sweden; ^4^ Translational Science and Experimental Medicine, Research and Early Development, Cardiovascular Renal and Metabolism (CVRM), BioPharmaceutical R&D AstraZeneca, Gothenburg, Sweden; ^5^ Drug Metabolism and Pharmacokinetics, Research and Early Development, Cardiovascular Renal and Metabolism (CVRM), BioPharmaceutical R&D AstraZeneca, Gothenburg, Sweden; ^6^ Institute for Artificial Intelligence, Russian Technological University (MIREA), Moscow, Russia; ^7^ I.M. Sechenov First Moscow State Medical University, Moscow, Russia

**Keywords:** BCAA, BCKA, BCKDK, BT2, cardiac hypertrophy, quantitative systems pharmacology, Model

## Abstract

**Background:** Abnormal branched-chained amino acids (BCAA) accumulation in cardiomyocytes is associated with cardiac remodeling in heart failure. Administration of branched-chain α-keto acid dehydrogenase (BCKD) kinase inhibitor BT2 has been shown to reduce cardiac BCAA levels and demonstrated positive effects on cardiac function in a preclinical setting. The current study is focused on evaluating the impact of BT2 on the systemic and cardiac levels of BCAA and their metabolites as well as activities of BCAA catabolic enzymes using a quantitative systems pharmacology model.

**Methods:** The model is composed of an ordinary differential equation system characterizing BCAA consumption with food, disposal in the proteins, reversible branched-chain-amino-acid aminotransferase (BCAT)-mediated transamination to branched-chain keto-acids (BCKA), followed by BCKD-mediated oxidation. Activity of BCKD is regulated by the balance of BCKDK and protein phosphatase 2Cm (PP2Cm) activities, affected by BT2 treatment. Cardiac BCAA levels are assumed to directly affect left ventricular ejection fraction (LVEF). Biochemical characteristics of the enzymes are taken from the public domains, while plasma and cardiac BCAA and BCKA levels in BT2 treated mice are used to inform the model parameters.

**Results:** The model provides adequate reproduction of the experimental data and predicts synchronous BCAA responses in the systemic and cardiac space, dictated by rapid BCAA equilibration between the tissues. The model-based simulations indicate maximum possible effect of BT2 treatment on BCAA reduction to be 40% corresponding to 12% increase in LVEF. Model sensitivity analysis demonstrates strong impact of BCKDK and PP2Cm activities as well as total BCKD and co-substrate levels (glutamate, ketoglutarate and ATP) on BCAA and BCKA levels.

**Conclusion:** Model based simulations confirms using of plasma measurements as a marker of cardiac BCAA changes under BCKDK inhibition. The proposed model can be used for optimization of preclinical study design for novel compounds targeting BCAA catabolism.

## Introduction

Branched-chained amino acids (BCAA)—leucine (Leu), isoleucine (Ile) and valine (Val) are essential amino acids obtained by food intake (Harper et al., 1984). BCAA are used as precursors for *de novo* protein synthesis or for energy supply (Neinast et al., 2019). In addition, BCAA have various biological effects including anabolic activity, suppressive effect on the insulin signaling and activation of the insulin and glucagon production (Hutson et al., 2005; Holeček, 2018; Wada et al., 2021). BCAA also plays a role in the neural system by donating nitrogen for the synthesis of neurotransmitters glutamate and γ-amino butyric acid (Hutson et al., 2005).

**FIGURE 1:**
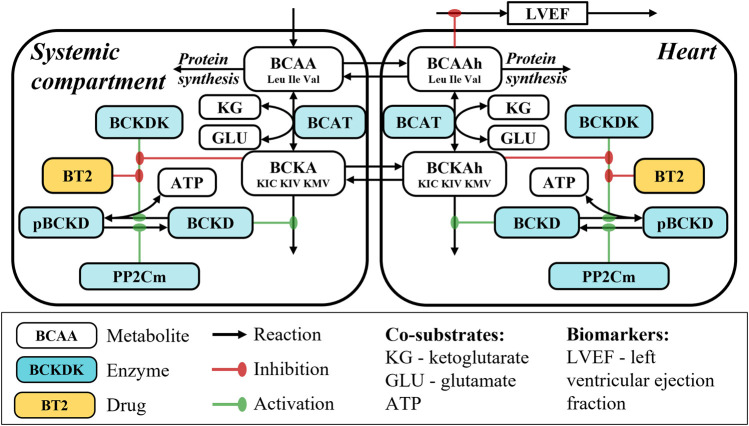
Model schematics.

BCAA oxidation is a multistep process that starts with reversible BCAA deamination by branched-chain-amino-acid aminotransferase (BCAT) enzyme and formation of branched-chain keto acids (BCKA)–alpha-ketoisocaproic (KIC), alpha-ketoisovaleric (KIV), and alpha-keto-beta-methylvaleric (KMV) acid. This step requires α-ketoglutarate and glutamate as co-substrates for the forward and reverse reaction, respectively. The BCKA undergo further irreversible oxidative decarboxylation catalyzed by the branched-chain α-keto acid dehydrogenase (BCKD) complex localized on the inner surface of mitochondrial membrane (Suryawan et al., 1998; Shimomura et al., 2001; Shimomura et al., 2004). Activity of BCKD enzyme is tightly regulated by a phosphorylation-dephosphorylation mechanism, affected by the delicate balance between the BCKD kinase (BCKDK) and protein phosphatase 2Cm (PP2Cm) activities (Shimomura et al., 2001) (Figure 1).

Distribution of BCAT and BCKD enzymes is shown to significantly vary between the tissues with the highest transamination and oxidation capacities, calculated for muscle and liver tissues, respectively (Suryawan et al., 1998; Hutson et al., 2005). This spatial distribution of the enzymes results in interorgan exchange of BCAA and BCKA and enables nitrogen cycling within the body (Suryawan et al., 1998; Holeček, 2018; Walejko et al., 2021). Efficacy of intracellular BCAA transport to the mitochondrial space is suggested to be another limiting factor for BCAA oxidation rate. The recent study by Walejko et al. (2021) indicates that BCKA undergo reamination rather than oxidation process in rat cardiac tissue, which is hypothesized to be related to low expression of the mitochondrial BCAA transporter SLC25A44. In contrast to this data, Neinast et al. (2019) demonstrates significant BCAA oxidation and limited BCAA disposal into proteins in mouse heart. These discrepancies can be attributed to the between-species differences in the enzyme expression levels, reported previously (Suryawan et al., 1998).

Whereas BCAA are being extensively used as food supplements and have been tested as a potential treatment option for cachexia and liver diseases (Holeček, 2018), abnormalities in BCAA catabolism are associated with metabolic disorders (Jørgenrud et al., 2015; Arany and Neinast, 2018; Biswas et al., 2020). In diabetic mouse models, plasma BCAA levels are increased whereas expression of BCAA catabolizing enzymes in the liver and the pancreatic islets are downregulated (Arany and Neinast, 2018; Wada et al., 2021). Intravenous BCAA infusions are shown to decrease insulin sensitivity and increase endogenous glucose production in humans which can be related to their effect on the insulin signaling and glucagon production (Krebs et al., 2002; Krebs et al., 2003; Uddin et al., 2019).

Pre-clinical animal models and clinical data indicate BCAA involvement in the cardiac remodeling process. Accumulation of BCAA and decrease in expression of BCAA catabolic enzymes in the cardiac tissue are observed in preclinical models of myocardial infarction [coronary artery ligation (CAL)] and heart failure [transverse aortic constriction (TAC)] (Wang et al., 2016; Uddin et al., 2019; Li et al., 2020) as well as in patients with dilated cardiomyopathy (Uddin et al., 2019). Cardiac BCAA accumulation is hypothesized to contribute to myocardial hypertrophy *via* mTOR-dependent activation of the protein synthesis and is shown to downregulate cardiac fatty acids and glucose oxidation (Huang et al., 2011; Li et al., 2017; Li et al., 2020). Given existing experimental evidence indicating BCAA accumulation in the cardiac, pancreatic, and hepatic tissue, and the association of BCAA levels with obesity and metabolic traits, targeting BCAA catabolism represents a conceivable strategy for the treatment of metabolic and cardiac diseases.

Among possible therapeutic options targeting the BCAA catabolic pathway, BCKDK inhibitors are being actively investigated. Endogenous BCKDK inhibition by BCKA was discovered by Paxton and Harris and represents regulatory feedback activating BCKA catabolism in case of their accumulation (Paxton and Harris, 1984). Allosteric BCKDK inhibitor BT2, identified in 2014 using high-throughput screening by Tso et al. (2013); Tso et al. (2014), has been shown to reduce BCAA levels in the systemic circulation and cardiac tissue and demonstrated efficacy in the myocardial infraction and diabetes models in preclinical settings (Chen et al., 2019; Uddin et al., 2019; Zhou et al., 2019; Lian et al., 2020). However, the complex network of feedbacks underlying the regulations of BCAA catabolism as well as the aspects of drug and target distribution substantiate the need for further quantitative research of potential factors influencing the efficacy of BCKDK inhibitors.

Quantitative systems pharmacology (QSP) is a relatively new modeling discipline, stemming from the roots of systems biology and used to get a mechanistic understanding of the systems dynamics and the effects of pharmacological interventions (NIH Workgroup, 2011). In contrast to the empirical pharmacokinetic/pharmacodynamic (PK/PD) models, QSP model is based on the principles of chemical and enzymological kinetics, homeostatic processes, and multicompartmental structure mimicking physiological distribution of the biological entities (Bradshaw et al., 2019). In this work, a first-in-class QSP model of BCAA catabolism was developed to characterize the dynamic interplay between multiple biological factors (e.g., enzyme activities, concentrations of the co-substrates) and the effect of BT2 inhibition on BCAA concentrations in different compartments. The model serves as a guide for the future preclinical studies and as a basis for the succeeding iterations of the mathematical modeling of the system under investigation.

## Materials and methods

### Experimental data

Data from six experiments on healthy female C57Bl6 mice 8–10 weeks old (C57BL/6NCrl Charles River, Germany) were available. Male mice were excluded for practical and ethical reasons: female mice can be housed in group while male mice need to be housed individually to avoid fighting. All animal studies were performed according to AstraZeneca Institutional Animal Care and Use Committee guidelines. BT2 was formulated in 0.5% HPMC 0.1% Tween. Animals were treated with vehicle only, or a single oral dose of 15, 40 or 120 mg/kg or two oral 40 mg/kg doses of BT2 given once daily. Plasma levels of BCAA, BCKA and BT2 were measured using mass spectrometry at baseline and 20 min or 1, 4, 7, 24, 48 h after dosing (Supplementary Table S1). Cardiac BCAA and BCKA measurements were available for the vehicle group, measured 48 h post dose. Detailed description of the experimental assay procedures is available in the Supplementary Material.

### Model structure

The model is represented by a system of ordinary differential equations reflecting the turnover of individual BCAA (Leu, Ile, Val) through the description of BCAA consumption with food, BCAA and BCKA exchange between systemic circulation and heart tissue, systemic and cardiac catabolism as well as BCAA usage for the protein synthesis.

Contribution of each organ to the systemic BCAA catabolism is not considered in the model and the respective systemic reaction rates therefore represent cumulative catabolic tissue capacities. The processes of BCAA catabolism in the heart are mirrored in the systemic circulation and are represented by BCAT-mediated reversible BCAA transamination followed by BCKD-mediated oxidative decarboxylation of BCKA. Activity of BCKD is regulated by BCKDK-mediated phosphorylation and PP2Cm-dependent dephosphorylation. In turn, activity of BCKDK is negatively affected by allosteric inhibitors KIC and BT2. Plasma BT2 dynamics is characterized using a standard 1-compartment pharmacokinetic (PK) model with first-order linear absorption and elimination and dose-dependent bioavailability, with rapid equilibration between systemic circulation and cardiac tissue.

The model structure is reported in Figure 1. List of the model equations as well as derivation of reaction rates is available in the Supplementary Material.

### Model calibration

The model includes 65 parameters taken from the publicly available sources or verified based on the experimental data. Kinetic parameters characterizing BCAT, BCKD, BCKDK, and PP2Cm activities and substrate specificities were taken from the respective studies (Pettit et al., 1978; Paxton and Harris, 1984; Yennawar et al., 2006; Wynn et al., 2012). Levels of ketoglutarate and ATP were assumed to be constant over time and fixed based on the experimental data (Ogawa et al., 2014). Daily BCAA intake was calculated based on the daily food composition and food content information. Rate constants, characterizing BCAA and BCKA exchange between plasma and cardiac tissue, were set to values enabling rapid tissue equilibration of the metabolites in line with observations (Neinast et al., 2019). Parameters quantifying BCAA conversion to proteins, BCKDK to PP2Cm ratio and glutamate concentration were expressed using values of other parameters and experimental data (equations are available in Supplementary Material).

The remaining model parameters were calibrated against the available experimental data using a sequential modeling approach. First, PK parameters characterizing plasma BT2 dynamics were estimated using plasma BT2 measurements. Second, placebo-adjusted plasma BCAA and BCKA levels were used to quantify systemic parameters of BCAA catabolism as well as pharmacodynamic parameters, responsible for the inhibitory effect of BT2 on BCKDK. Third, parameters of cardiac BCAA catabolism as well as BCKA efflux from cardiac tissue to plasma were estimated using cardiac BCAA and BCKA measurements in healthy animals. Parameters characterizing total BCKD and BCKDK to PP2Cm ratio in the systemic circulation were found to be interdependent, thus, total BCKD level was fixed.

To reproduce BCAA catabolism in heart failure conditions, model parameters characterizing protein synthesis as well as PP2Cm and BCAT activities in cardiac tissue were modified in line with experimental evidence generated in TAC mice (Sun et al., 2016). Finally, the link between cardiac BCAA changes and cardiac status was quantified using LVEF dynamics following TAC surgery and BT2 or vehicle treatment.

The summary of the model parameters can be found in the Supplementary Table S2.

### Model based simulations

To perform sensitivity analysis values of the selected model parameters were derived using Latin Hypercube Sampling (LHS): parameter values were randomly generated in 0.1–10-fold range from their estimates and used to predict effect of 40 mg/kg daily BT2 dosing on various model variables. Pairwise partial correlations between parameter values and biomarkers were estimated using Spearman method (Iman et al., 1981).

### Software

The model development and parameter estimation were performed in Monolix^®^ 2019R1 software (Lixoft, Antony, France). Exploratory analysis and visualization of the data and the model simulations were performed in R computing environment (R version 3.5.1) using tidyverse (version 1.3.1) and RsSimulx (version 1.0.1) packages.

## Results

### The model captures the experimental data

**FIGURE 2:**
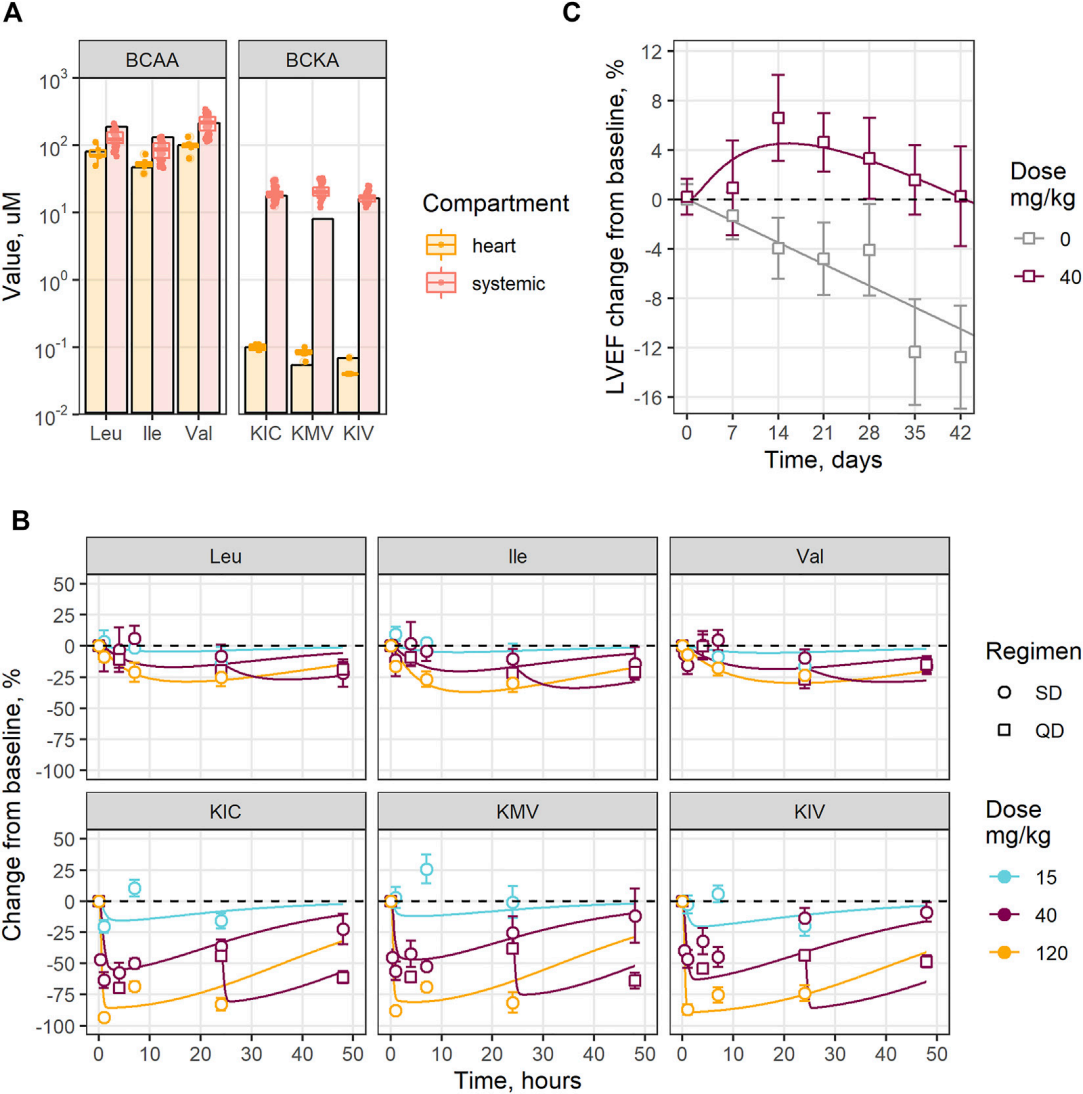
Experimental data reproduction by the model: **(A)** Steady-state BCAA and BCKA levels; **(B)** Change over time in plasma BCAA and BCKA levels under different BT2 doses and regimens (SD—single dose, QD—once daily); **(C)** LVEF change in mouse after TAC surgery, treated with vehicle or BT2. Circles, squares and error bars—observed mean values and standard errors, lines—model predictions; dots—individual measurements in plasma, bars—model predictions.

The developed QSP model of BCAA catabolism adequately reproduced the experimental data generated in healthy and TAC mice (Figure 2, Supplementary Figure S3). Baseline BCAA concentrations were approximately 5-fold and 500-fold higher compared to the BCKA in the systemic circulation and cardiac tissue, respectively (Figure 2A). Larger difference between BCKA and BCAA concentrations in the heart relative to the systemic exposure is associated with the higher activity of BCKD in the former, as shown in the parameter estimates (Supplementary Table S2). The model-based simulations indicate a decrease in active BCKD due to the change in BCKDK to PP2Cm ratio and showed more pronounced increase in BCKA vs*.* BCAA levels in the heart in a TAC state (Supplementary Figure S4).

The model captures dose-dependent reduction in the systemic BCAA and BCKA levels under BT2 treatment, with observed maximum inhibitory effect of −25% for Leu, Ile, Val, and of −25% for KIC, KMV, KIV, respectively (Figure 2B). Treatment-mediated changes in BCAA catabolism were associated with an approximately 20% increase in LVEF compared to the placebo in mice subjected to TAC surgery, in both the model and the observed data, reaching maximum benefit after 2 weeks of treatment (Figure 2C) (Tso et al., 2013).

### Dose-dependent BT2 effect on metabolites and enzyme activities

**FIGURE 3:**
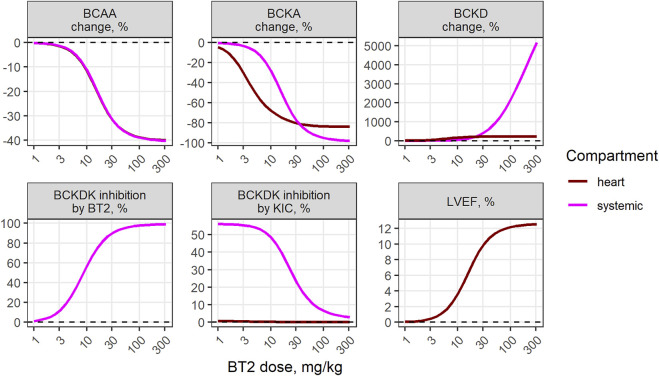
Model-predicted dose-dependent BT2 effect on various markers of BCAA catabolism in the systemic circulation and the cardiac tissue.

The mechanistic model was used to quantify baseline states and dose-response relationships between BT2 and the variety of cardiac and BCAA-related markers in the heart, as well as in systemic circulation. According to the model structure and underlying physiology, BCKD activity, mediated by BCKDK and PP2Cm, is negatively affected by BT2 and BCKA (Figure 3). At baseline, approximately 50% of the systemic BCKDK is inhibited by KIC, whereas the contribution of KIC to BCKDK inhibition in cardiac tissue is minor; the latter fact is explained by the lower KIC levels in the cardiac vs*.* the systemic compartment. The BT2 treatment decreases KIC concentration, thereby indirectly stimulating BCKDK activity. However, this effect is completely overshadowed by the BT2 inhibition of BCKDK itself, in a dose-dependent manner.

Model-based simulations demonstrate matching BCAA reduction relative to the dose between plasma and cardiac tissue, with approximately 40% maximum treatment effect and was similar between healthy and TAC animals (Figure 3, Supplementary Figure S4). The associated maximum benefit in LVEF was estimated to be 12%. Predicted maximum inhibition of cardiac BCKA levels was less pronounced than that of plasma BCKA, with 80% and 100% reduction, respectively.

### Homeostasis and perturbations of the system

**FIGURE 4:**
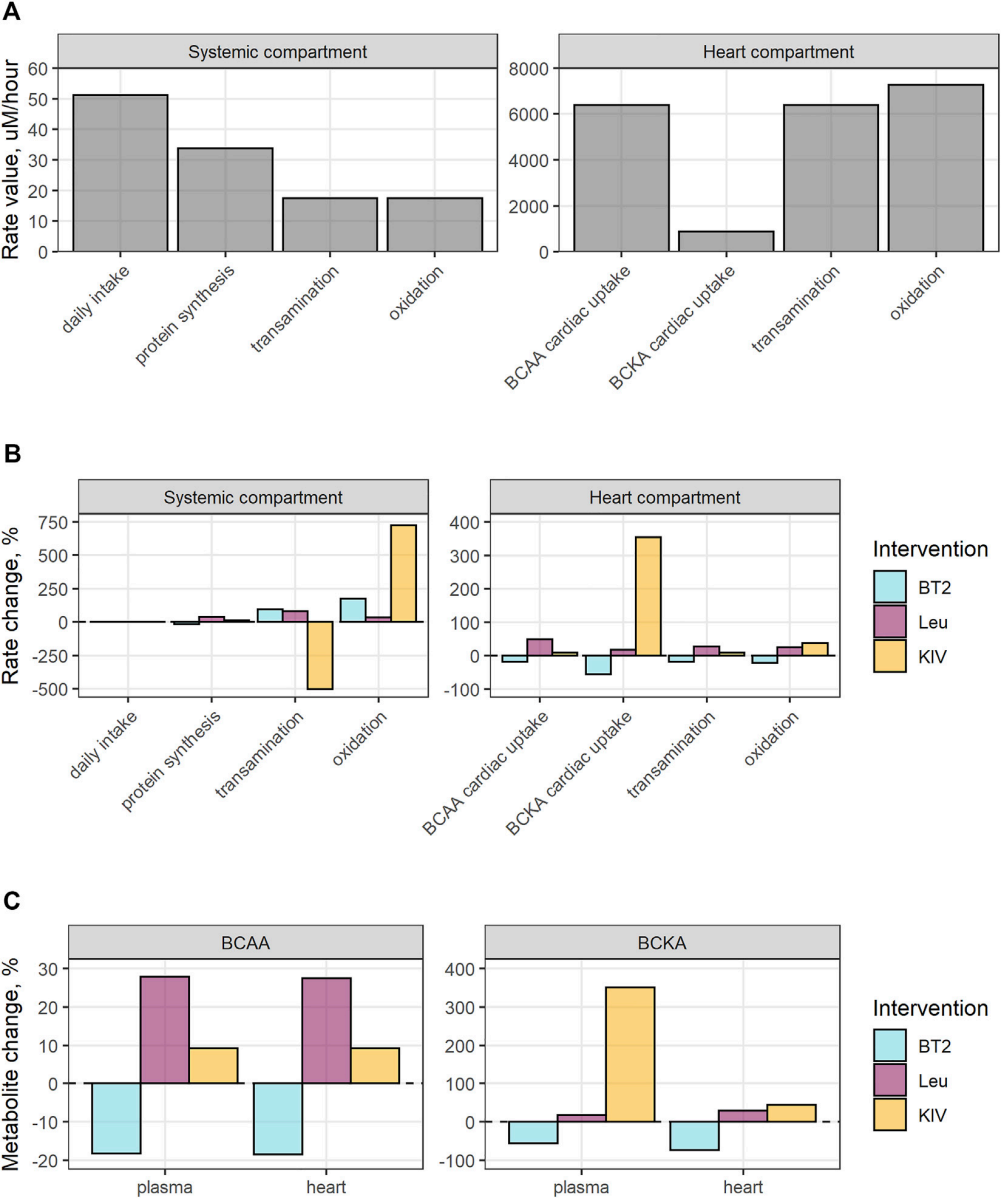
Analysis of the impact of BT2, BCAA or BCKA intake on the homeostatic system. **(A)** Pre-treatment reaction rates; **(B)** The effect of interventions on the reaction rates; **(C)** Changes in metabolite levels under the treatment. Simulation design implied single administration of 40 mg/kg BT2, 100 umol Leu, or 100 umol KIV; maximal impact of the treatment on biomarkers is shown.

To gain better understanding of systemic and cardiac BCAA catabolism we performed quantitative analysis of the BCAA and BCKA transamination, oxidation, and transport rates, affecting BCAA and BCKA levels in plasma and cardiac tissue, with and without interventions (Figure 4).

The homeostatic nature of the system is ensured at the pre-treatment condition by the complex equilibrium of multiple rates: daily BCAA intake is equal to the sum of BCAA conversion to proteins and BCAA transamination rates, with the latter being equal to BCKA oxidation rate. Cardiac BCAA transamination is compensated by the BCAA uptake from the systemic plasma to the heart, while cardiac BCKA oxidation is balanced out by BCAA transamination and BCKA uptake (Figure 4A). Given the reversibility of the transamination reaction, *de novo* BCAA synthesis from BCKA in cardiac tissue is possible but is not predicted by the model at baseline condition.

BT2 treatment activates the oxidation of BCKA, shifting the equilibrium towards the BCAA transamination into BCKA, leading to the reduction in systemic BCAA and a decrease in the protein synthesis. Resulting drop in the cardiac uptake of both BCAA and BCKA is reflected in the decrease of cardiac transamination and oxidation rates (Figure 4B). Conversely, additional daily intake of BCAA and BCKA results in an increase in cardiac concentrations of the respective entities and the compensatory changes in transamination rates (Figures 4B,C).

### Systems analysis of the BCAA catabolism and cardiac function

**FIGURE 5:**
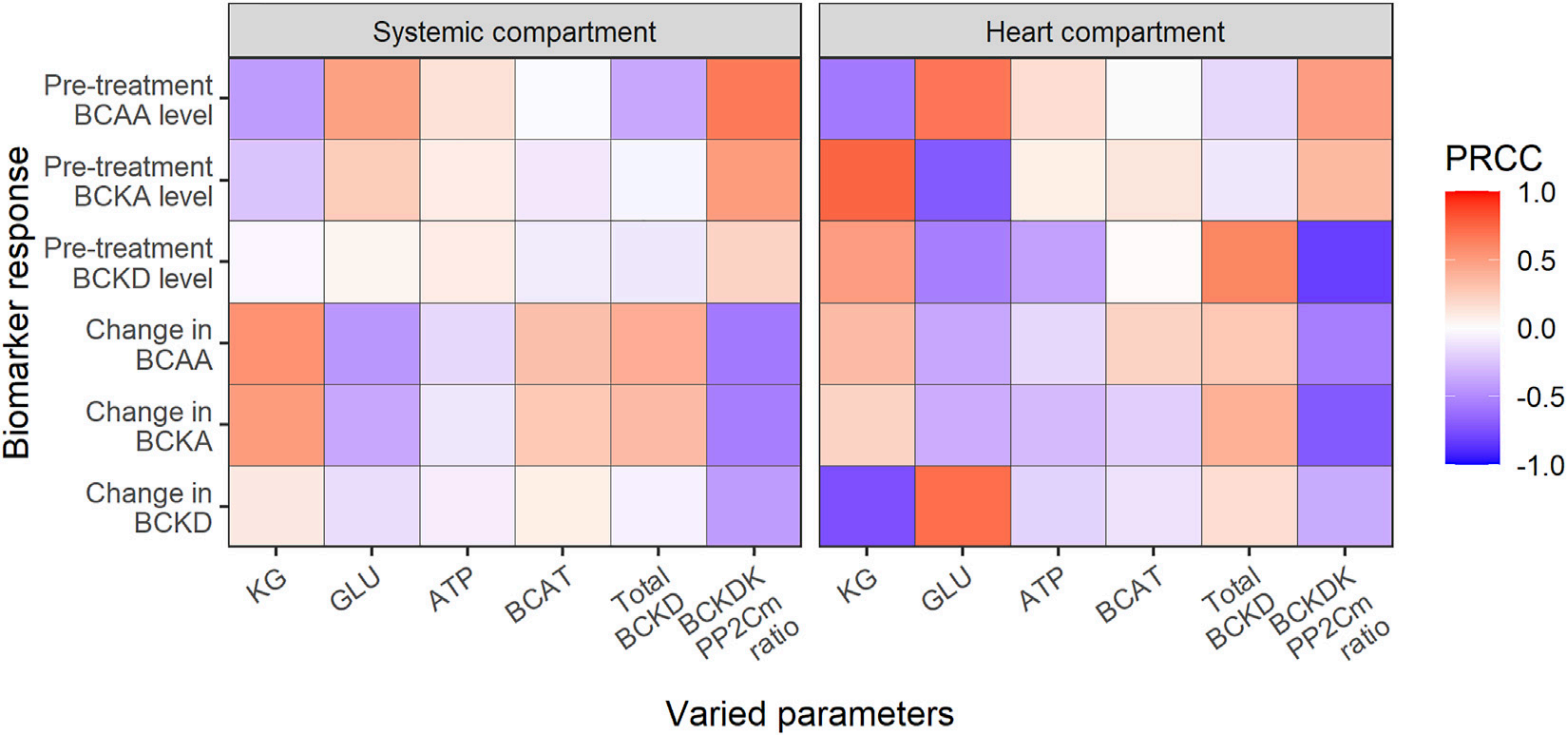
Global sensitivity analysis using partial rank correlation coefficient (PRCC). Heatmap reflects the degree of association between parameters and biomarkers either at baseline (pre-treatment) or at treatment steady-state (change) after daily BT2 administration. Red and blue color denotes positive and negative association, respectively; KG—ketoglutarate, GLU—glutamate.

The mechanistic origin of the model allows to explore the contribution of various factors within the network of BCAA catabolism (e.g., co-substrate and enzyme concentrations) on both baseline state and the magnitude of BT2 effect, through the process of sensitivity analysis (Figure 5). The model equations did not include cardiac BCAA and BCKA effect on the systemic BCAA and BCKA levels and therefore variation of the parameters characterizing cardiac BCAA catabolism did not cause changes in the plasma biomarkers.

Concentrations of ketoglutarate (KG), glutamate (GLU) and ATP co-substrates, BCAT and BCKD enzymes as well as BCKDK/PP2Cm ratio were varied within the pre-defined range while the changes in BCAA, BCKA and BCKD levels (pre-treatment and after BCKDK inhibition) were evaluated.

All co-substrates were found to have a significant effect on the levels of amino acids, among them KG and GLU having most prominent effect. As KG is positively linked with BCAA deamination rate, high KG concentrations result in a decrease in baseline BCAA levels, accumulation of BCKA, and activation of BCKD. Inversely, GLU is negatively associated with the reaction and has the opposite effect on BCAA and BCKA levels. ATP activates BCKD phosphorylation leading to the minor increase in BCKA and BCAA levels through the reduction of BCKA oxidation rate.

As such, in contrast to KG, high ATP or GLU interfere with the BCKDK inhibition and decrease overall net benefit of the BT2 treatment.

The effects of BCAT, total BCKD as well as BCKDK/PP2Cm ratio variations on BT2 efficacy were different. Changes in BCAT activity did not alter baseline BCAA and BCKA levels, as the transamination rate keeps the model in the state of equilibrium (Holeček, 2018). However, the increased BCAT activity combined with BT2 treatment boosts the forward transamination rate, resulting in an increased treatment benefit in BCAA. An increase in the total BCKD pool negatively affects the steady-state BCAA and BCKA concentrations, limiting the efficacy of BT2. Similarly, the BCKDK/PP2Cm ratio strongly affected the BCKD activity, resulting in the accumulation of BCAA and BCKA and decrease in the BT2 treatment effects.

## Discussion

In our current research, we report the development and application of the mechanistic mathematical model of BCAA catabolism used to reconstruct and investigate the dose-response relationship of BT2 (a BCKDK inhibitor) efficacy in mice. The proposed multiscale model is the first of its kind and integrates 1) the information on BCAT, BCKD, BCKDK and PP2Cm enzymes (e.g. substrate specificities, catalytic constants), which was extracted from the earlier studies (Pettit et al., 1978; Paxton and Harris, 1984; Yennawar et al., 2006; Wynn et al., 2012), 2) measurements of BCAA and BCKA fluxes in the body reported by Neinast et al. (2019) and 3) *de novo* generated BT2 pharmacokinetic and pharmacodynamic data.

First step in model development was to characterize systemic BCAA catabolism. It is known that BCAA-related catabolic processes are spatially separated and take place in different organs, with the skeletal muscle and the liver being the key sites of BCAA transamination and BCKA oxidation, respectively (Holeček, 2018). However, the amount of the quantitative information on the BCAA, BCKA and co-substrates levels in different organs and the rates of interorgan exchange is limited. As such, in the proposed model systemic BCAA catabolism is not compartmentalized to avoid numerous assumptions that can substantially impair predictive power of the model.

While BT2 effect on plasma BCAA level has been evaluated previously (Chen et al., 2019; Lian et al., 2020; Walejko et al., 2021), only 20, 40 and 50 mg/kg doses have been tested in these experiments. Model-based analysis of the newly generated and historical data reveals maximal treatment benefit of BT2 to be achieved with the oral dose of 120 mg/kg, which can serve as a point of reference for the future tests in preclinical models of heart failure. According to our results, oral BT2 dose of 15 mg/kg does not lead to a significant BCAA and BCKA response in plasma, whereas intraperitoneal (i.p.) once-daily injections of 20 mg/kg BT2 led to significant reduction in cardiac BCAA levels, as reported in the literature (Wang et al., 2016; Lian et al., 2020), which can be explained by possible differences in BT2 pharmacokinetics following oral and intraperitoneal drug administration.

Calibration of the cardiac BCAA catabolism was complicated by the contradictory experimental data available for the model development but was necessary to evaluate BT2 effect on cardiac functional readouts such as LVEF. Significant differences in baseline cardiac BCAA and BCKA levels between published experiments were identified (Sun et al., 2016; Chen et al., 2019; Uddin et al., 2019) with the highest biomarker values observed Chen et al. (2019). These measurements were 10-fold higher compared to the data generated by our team and findings by Sun et al. (2016). We hypothesized this discrepancy to be a consequence of possible differences in the mouse diet as well as extraction and measuring methodologies. Our data indicate slightly lower BCAA levels and approximately 100-fold lower BCKA levels in cardiac tissue vs*.* plasma.

To characterize enzyme activities in the cardiac tissue we used not only cardiac BCAA and BCKA measurements but also included information about reaction rates. Data from (Neinast et al., 2019) highlights rapid equilibration between plasma and organ BCAA and BCKA levels in the experiments with BCAA and BCKA infusion, respectively, which was reflected in the model parameters. Calculations performed by Neinast et al. (2019) indicated 4% of total BCAA oxidation takes place in the heart. Given relatively small heart volume, calculated cardiac oxidation rate per heart volume unit is very fast, which can explain low BCKA levels in cardiac tissue. In contrast, recent data from (Walejko et al., 2021), generated in the perfused rat hearts point towards BCKA reamination rather than oxidation being a predominant process. Information from both studies could not be reproduced by the model simultaneously, therefore, we focused on the study (Neinast et al., 2019), given the similarities in the experimental design.

Discrepancies in BT2 impact on cardiac BCAA and BCKA were also observed between the previously reported studies. For example, 20 mg/kg once daily intraperitoneal BT2 dosing was shown to reduce cardiac BCAA by 50% in the experiment (Wang et al., 2016) whereas other experiment demonstrated 40 mg/kg BT2 treatment resulting only in 30% decrease in BCAA (Uddin et al., 2019). Given poor BT2 solubility we hypothesize these differences to be related to the variations in drug bioavailability across the experiments, however, the BT2 pharmacokinetic measurements were not reported to confirm or decline this hypothesis. Nonetheless, all identified publications confirmed significant BCAA and BCKA reduction under BT2 treatment (by approximately 27%–45% and 13%–50%, respectively) (Wang et al., 2016; Chen et al., 2019; Uddin et al., 2019; Lian et al., 2020). Model-based simulations indicated 35% and 80% decrease in cardiac BCAA and BCKA levels under 40 mg/kg oral BT2 treatment, BCAA response between plasma and heart being very similar, therefore substantiating plasma BCAA measurements as sufficient for evaluation of the BT2 treatment activity.

Interestingly, the model analysis revealed a strong connection between systemic BCAA and BCKA and the cardiac amino- and ketoacids response to different interventions, i.e., the BT2 effect on cardiac BCAA and BCKA levels is dependent on the systemic BCAA and BCKA reduction rather than on local inhibitory effect on cardiac BCKDK, which is in line with the hypothesis proposed by Walejko et al. (2021). Neinast and others did not observe and increase in cardiac BCKA oxidation under BT2 despite pronounced activation of cardiac BCKD, whereas our predictions indicate small reductions in cardiac BCKA oxidation following BT2-mediated reduction of cardiac BCAA and BCKA uptake. Model-based simulations without the systemic BT2 effect (Supplementary Figure S4) indicated no significant changes in cardiac BCKA oxidation despite the notable increase in BCKD activity, which can be attributed towards the low cardiac BCKA level limiting oxidation process.

Following these findings, systemic administration of BCAA or BCKA (e.g. with food supplements) affect not only systemic but also cardiac BCAA catabolism. No information about negative effects of BCAA intake on cardiac functioning was found in the literature. However, observations from patients with defected BCAA catabolism, where systemic and cardiac BCAA and BCKA accumulation negatively impacted the cardiac function (Huang et al., 2011), support the claim that supraphysiological BCAA intake might have a detrimental effect on heart failure in patients with the defective BCAA catabolism, but not in healthy individuals.

Although the model is built on rodent data, it could potentially be used to inform human dose prediction for possible drug candidates. While keeping similar model structure, parameters related to the kinetics of a drug, expression of the enzyme levels as well as BCAA intake would potentially have to be scaled in a suitable manner (Suryawan et al., 1998). Effect size on functional parameters (i.e. LVEF) would be complicated to scale as very little human data are available on the relationship but could possibly be calibrated in early clinical studies to allow model informed design in later studies.

## Conclusion

We developed a first-in-class multi-scale systems pharmacology model of BCAA catabolism that integrates the existing knowledge on the enzyme kinetics with the pool of heterogeneous preclinical data to not only quantitatively characterize the changes in BCAA and BCKA levels in response to the BCKDK inhibition across physiological compartments, but also to gain better understanding of the underlying homeostatic system and mechanisms of drug action as well as to interpret observed data in mice. The model supports the hypothesis of systemic BCAA measurements being representative of the cardiac levels of the amino acids, deconvolutes the intricate interplay between the markers of BCAA catabolism to reconstruct dose-response of the BT2 inhibitor and quantifies the impact of the confounding factors (e.g., co-substrate concentrations) on the relationship *via* a sensitivity analysis. The proposed model can serve as a useful tool for evaluation of *in vivo* activity of novel BCKDK inhibitors and other compounds affecting BCAA catabolism as well as optimization of preclinical study design, informing sampling, and dosing schedules. The model can also be used for mechanistic hypothesis generation to suggest further experiments for elucidation of the system.

## Data Availability

The data that support the findings of this study are available from the corresponding author upon reasonable request.

## References

[B1] Arany Z. Neinast M. (2018). Branched chain amino acids in metabolic disease. Curr. Diab. Rep. 18 (10), 76. 10.1007/s11892-018-1048-7 30112615

[B2] Biswas D. Tozer K. Dao K. T. Perez L. J. Mercer A. Brown A. (2020). Adverse outcomes in obese cardiac surgery patients correlates with altered branched-chain amino acid catabolism in adipose tissue and heart. Front. Endocrinol. 11, 534. 10.3389/fendo.2020.00534 PMC7438793 32903728

[B3] Bradshaw E. L. Spilker M. E. Zang R. Bansal L. He H. Jones R. D. O. (2019). Applications of quantitative systems pharmacology in model-informed drug discovery: Perspective on impact and opportunities. CPT. Pharmacometrics Syst. Pharmacol. 8 (11), 777–791. 10.1002/psp4.12463 31535440 PMC6875708

[B4] Chen M. Gao C. Yu J. Ren S. Wang M. Wynn R. M. (2019). Therapeutic effect of targeting branched‐chain amino acid catabolic flux in pressure-overload induced heart failure. J. Am. Heart Assoc. 8, e011625. 10.1161/JAHA.118.011625 31433721 PMC6585363

[B5] Harper A. E. Miller R. H. Block K. P. (1984). Branched-chain amino acid metabolism. Annu. Rev. Nutr. 4, 409–454. 10.1146/annurev.nu.04.070184.002205 6380539

[B6] Holeček M. (2018). Branched-chain amino acids in health and disease: Metabolism, alterations in blood plasma, and as supplements. Nutr. Metab. 15 (1), 33. 10.1186/s12986-018-0271-1 PMC5934885 29755574

[B7] Huang Y. Zhou M. Sun H. Wang Y. (2011). Branched-chain amino acid metabolism in heart disease: An epiphenomenon or a real culprit? Cardiovasc. Res. 90 (2), 220–223. 10.1093/cvr/cvr070 21502372 PMC3078803

[B8] Hutson S. M. Sweatt A. J. Lanoue K. F. (2005). Branched-chain [corrected] amino acid metabolism: Implications for establishing safe intakes. J. Nutr. 135 (6), 1557S–64S. 10.1093/jn/135.6.1557S 15930469

[B9] Iman R. L. Helton J. C. Campbell J. E. (1981). An approach to sensitivity analysis of computer models: Part I—introduction, input variable selection and preliminary variable assessment. J. Qual. Technol. 13 (3), 174–183. 10.1080/00224065.1981.11978748

[B10] Jørgenrud B. Jalanko M. Heliö T. Jääskeläinen P. Laine M. Hilvo M. (2015). The metabolome in Finnish carriers of the MYBPC3-q1061x mutation for hypertrophic cardiomyopathy. PLOS ONE 10 (8), e0134184. 10.1371/journal.pone.0134184 26267065 PMC4534205

[B11] Krebs M. Brehm A. Krssak M. Anderwald C. Bernroider E. Nowotny P. (2003). Direct and indirect effects of amino acids on hepatic glucose metabolism in humans. Diabetologia 46 (7), 917–925. 10.1007/s00125-003-1129-1 12819901

[B12] Krebs M. Krssak M. Bernroider E. Anderwald C. Brehm A. Meyerspeer M. (2002). Mechanism of amino acid-induced skeletal muscle insulin resistance in humans. Diabetes 51 (3), 599–605. 10.2337/diabetes.51.3.599 11872656

[B13] Li T. Zhang Z. Kolwicz S. C. Abell L. Roe N. D. Kim M. (2017). Defective branched-chain amino acid catabolism disrupts glucose metabolism and sensitizes the heart to ischemia-reperfusion injury. Cell Metab. 25 (2), 374–385. 10.1016/j.cmet.2016.11.005 28178567 PMC5301464

[B14] Li Y. Xiong Z. Yan W. Gao E. Cheng H. Wu G. (2020). Branched chain amino acids exacerbate myocardial ischemia/reperfusion vulnerability via enhancing GCN2/ATF6/PPAR-α pathway-dependent fatty acid oxidation. Theranostics 10 (12), 5623–5640. 10.7150/thno.44836 32373236 PMC7196282

[B15] Lian K. Guo X. Wang Q. Liu Y. Wang R. T. Gao C. (2020). PP2Cm overexpression alleviates MI/R injury mediated by a BCAA catabolism defect and oxidative stress in diabetic mice. Eur. J. Pharmacol. 866, 866172796. 10.1016/j.ejphar.2019.172796 31738932

[B16] Neinast M. D. Jang C. Hui S. Murashige D. S. Chu Q. Morscher R. J. (2019). Quantitative analysis of the whole-body metabolic fate of branched-chain amino acids. Cell Metab. 29 (2), 417–429. e4. 10.1016/j.cmet.2018.10.013 30449684 PMC6365191

[B17] NIH Workgroup (2011). Quantitative and systems pharmacology in the post-genomic era: New approaches to discovering drugs and understanding therapeutic mechanisms. Bethesda.

[B18] Ogawa T. Washio J. Takahashi T. Echigo S. Takahashi N. (2014). Glucose and glutamine metabolism in oral squamous cell carcinoma: Insight from a quantitative metabolomic approach. Oral Surg. Oral Med. Oral Pathol. Oral Radiol. 118 (2), 218–225. 10.1016/j.oooo.2014.04.003 24927638

[B19] Paxton R. Harris R. A. (1984). Regulation of branched-chain alpha-ketoacid dehydrogenase kinase. Arch. Biochem. Biophys. 231 (1), 48–57. 10.1016/0003-9861(84)90361-8 6721501

[B20] Pettit F. H. Yeaman S. J. Reed L. J. (1978). Purification and characterization of branched chain alpha-keto acid dehydrogenase complex of bovine kidney. Proc. Natl. Acad. Sci. U. S. A. 75 (10), 4881–4885. 10.1073/pnas.75.10.4881 283398 PMC336225

[B21] Shimomura Y. Murakami T. Nagasaki M. Honda T. Goto H. Kotake K. (2004). Regulation of branched-chain amino acid metabolism and pharmacological effects of branched-chain amino acids. Hepatol. Res. 30, 3–8. 10.1016/j.hepres.2004.09.001 15607132

[B22] Shimomura Y. Obayashi M. Murakami T. Harris R. A. (2001). Regulation of branched-chain amino acid catabolism: Nutritional and hormonal regulation of activity and expression of the branched-chain α-keto acid dehydrogenase kinase. Curr. Opin. Clin. Nutr. Metab. Care 4 (5), 419–423. 10.1097/00075197-200109000-00013 11568504

[B23] Sun H. Olson K. C. Gao C. Prosdocimo D. A. Zhou M. Wang Z. (2016). Catabolic defect of branched-chain amino acids promotes heart failure. Circulation 133 (21), 2038–2049. 10.1161/CIRCULATIONAHA.115.020226 27059949 PMC4879058

[B24] Suryawan A. Hawes J. W. Harris R. A. Shimomura Y. Jenkins A. E. Hutson S. M. (1998). A molecular model of human branched-chain amino acid metabolism. Am. J. Clin. Nutr. 68 (1), 72–81. 10.1093/ajcn/68.1.72 9665099

[B25] Tso S. C. Gui W. J. Wu C. Y. Chuang J. L. Qi X. Skvora K. J. (2014). Benzothiophene carboxylate derivatives as novel allosteric inhibitors of branched-chain α-ketoacid dehydrogenase kinase. J. Biol. Chem. 289 (30), 20583–20593. 10.1074/jbc.M114.569251 24895126 PMC4110271

[B26] Tso S. C. Qi X. Gui W. J. Chuang J. L. Morlock L. K. Wallace A. L. (2013). Structure-based design and mechanisms of allosteric inhibitors for mitochondrial branched-chain α-ketoacid dehydrogenase kinase. Proc. Natl. Acad. Sci. U. S. A. 110 (24), 9728–9733. 10.1073/pnas.1303220110 23716694 PMC3683707

[B27] Uddin G. M. Zhang L. Shah S. Fukushima A. Wagg C. S. Gopal K. (2019). Impaired branched chain amino acid oxidation contributes to cardiac insulin resistance in heart failure. Cardiovasc. Diabetol. 18 (1), 86. 10.1186/s12933-019-0892-3 31277657 PMC6610921

[B28] Wada E. Kobayashi M. Kohno D. Kikuchi O. Suga T. Matsui S. (2021). Disordered branched chain amino acid catabolism in pancreatic islets is associated with postprandial hypersecretion of glucagon in diabetic mice. J. Nutr. Biochem. 97, 108811. 10.1016/j.jnutbio.2021.108811 34197915

[B29] Walejko J. M. Christopher B. A. Crown S. B. Zhang G. F. Pickar-Oliver A. Yoneshiro T. (2021). Branched-chain α-ketoacids are preferentially reaminated and activate protein synthesis in the heart. Nat. Commun. 12 (1), 1680. 10.1038/s41467-021-21962-2 33723250 PMC7960706

[B30] Wang W. Zhang F. Xia Y. Zhao S. Yan W. Wang H. (2016). Defective branched chain amino acid catabolism contributes to cardiac dysfunction and remodeling following myocardial infarction. Am. J. Physiol. Heart Circ. Physiol. 311 (5), H1160–H1169. 10.1152/ajpheart.00114.2016 27542406

[B31] Wynn R. M. Li J. Brautigam C. A. Chuang J. L. Chuang D. T. (2012). Structural and biochemical characterization of human mitochondrial branched-chain α-ketoacid dehydrogenase phosphatase. J. Biol. Chem. 287 (12), 9178–9192. 10.1074/jbc.M111.314963 22291014 PMC3308798

[B32] Yennawar N. H. Islam M. M. Conway M. Wallin R. Hutson S. M. (2006). Human mitochondrial branched chain aminotransferase isozyme: Structural role of the CXXC center in catalysis. J. Biol. Chem. 281 (51), 39660–39671. 10.1074/jbc.M607552200 17050531

[B33] Zhou M. Shao J. Wu C. Y. Shu L. Dong W. Liu Y. (2019). Targeting BCAA catabolism to treat obesity-associated insulin resistance. Diabetes 68 (9), 1730–1746. 10.2337/db18-0927 31167878 PMC6702639

