# Supplementary material for: Evaluation of therapeutic strategies targeting BCAA catabolism using a systems pharmacology model
Source: Front Pharmacol. 2022 Nov 28;13:993422. doi: 10.3389/fphar.2022.993422 (PMC9744226; doi:10.3389/fphar.2022.993422)
Supplement: Supplementary file 1 [file DataSheet2.DOCX]

Supplementary Material

# Experimental assays

## Compound analysis

BT2 in plasma was extracted by addition of 180 µL ice-cold acetonitrile containing 10 nM verapamil as internal standard, to 50 µL plasma. The samples were mixed at 600 rpm for 5 minutes, followed by centrifugation at 4000 rpm for 20 minutes at 4°C. A one-to-one dilution was subsequently performed by transferring 75 µL of the supernatant to 75 µL MilliQ water, followed by mixing at 600 rpm for 5 minutes. Extraction of BT2 from heart tissue involved an initial tissue homogenization step. 40-50 mg heart tissue was homogenized using 7 volumes (m/V) of Ringer’s Solution at 4°C. The homogenization was performed at 2500 rpm for 2x30s in reinforced 2 mL Precellys tubes containing 6 ceramic beads. 50 µL of the homogenate was then subjected to protein precipitation by addition of 180 µL of ice-cold acetonitrile containing 10 nM verapamil. Measurement of BT2 concentrations in both sample types was performed employing reverse phase ultra-high performance liquid chromatography (RP-UPLC) for sample deconvolution prior to analysis on a Waters TQ-S triple quadrupole mass spectrometer with an electrospray ionization interface. 1 µL sample was injected and subsequently separated over a Waters BEH C18 column (2.1, 50 mm; 1.7µ). Separation was achieved using a 1.7 min gradient starting at 96% H_2_0, 4% acetonitrile, 0.1% formic acid and ending at 4% H_2_0, 96% acetonitrile, 0.1% formic acid. BT2 in study samples was quantified by comparing the relative abundance of a single product ion to a linear regression model from an eight-point calibration curve prepared in parallel in blank matrix (plasma/heart-tissue from control animals), while simultaneous monitoring of two further fragments allowed for increased assay accuracy.

## BCAA and BCKA analysis

Plasma samples were extracted with methanol containing deuterated internal standards. After 10 minutes of vortex at 1400 rpm and centrifugation at 4000 rpm, 50µl of the supernatant was evaporated and reconstituted in 250µl water and analyzed.

Heart tissues (40-50mg) were homogenized in methanol containing deuterated internal standards using a Precellys instrument. The extraction was finalized using a TissueLyzer instrument (25Hz for 5 minutes). After centrifugation the supernatant (50µl) was evaporated. For BCAA analysis the supernatant was reconstituted in water and analyzed. For BCKA analysis the samples were derivatized prior to analysis according to previous work (1). BCAA and BCKA were measured using ultra-high performance liquid chromatography coupled to a Waters TQ-XS triple quadrupole (UPLC-MS/MS). Separation was achieved using a Waters BEH C8 column (2.1, 100mm; 1.7µ) with water (0.1% formic acid) and acetonitrile (0.1% formic acid) as mobile phases. The flow rate was 400µl per minute and the column was kept at 40 degrees. For plasma, detection was made in both positive (BCAA) and negative (BCKA) mode using intra-run polarity switching. For heart, two injections were made. One for the analysis of underivatized BCAA and one for the derivatized BCKA. Quantification was made using external standard curves (made in 20% methanol) that were prepared in parallel with the samples.

Additional information on BT2 treatment effect on left ventricular ejection fraction (LVEF) has been collected from study by Chen et al. (2). In the considered experiment C57BL/6N mice were subjected to TAC; once daily administration of vehicle or 40 mg/kg BT2 *via* oral gavage was initiated 2 weeks after the surgery and continued for 6 weeks. At the end of the experiment heart tissue was collected and BCAA and BCKA were measured as described in the paper (2).

# Model structure

The model captures dynamics of individual BCAA – leucine, isoleucine, valine (Leu, Ile, Val,) and BCKA - alpha-ketoisocaproic acid, alpha-ketoisovaleric, alpha-keto-beta-methylvaleric (KIC, KIV, KMV) species in the blood plasma and cardiac tissue. All rates are reported in concentration units.

## ODE system, capturing BCAA metabolism and exchange between the organs

Level of individual BCAA in plasma depends on the BCAA consumption with food ($Ra_{\mathrm{pl}}$), tissue BCAA deamination ($R{DAd}_{\mathrm{pl}}$) and BCAA to protein disposal rates ($Rd_{\mathrm{pl}})$ (eq. 1):

$\frac{d\mathrm{BCAA}_{\mathrm{pl}}}{\mathrm{dt}}=Ra_{\mathrm{pl}}-RDA_{\mathrm{pl}}-Rd_{\mathrm{pl}}$ (eq. 1)

Dynamics of individual BCKA in plasma depends on the balance between BCAA deamination rate ($Rd_{\mathrm{pl}}$) and BCKA oxidative decarboxylation rate ($\mathrm{Ro}x_{\mathrm{pl}}$) (eq. 2):

$\frac{d\mathrm{BCKA}_{\mathrm{pl}}}{\mathrm{dt}}=\mathrm{RD}A_{\mathrm{pl}}-Rox_{\mathrm{pl}}$ (eq. 2)

Cardiac BCAA level depends on the transport from the plasma (${Ra}_{heart}^{BCAA}{Ra}_{heart}$) and deamination rates ($\mathrm{RD}A_{\mathrm{heart}}$); minor BCAA fraction is used for the protein synthesis in the heart tissue (3) and therefore this process was not taken into account in the model (eq.3):

$\frac{d\mathrm{BCAA}_{\mathrm{heart}}}{\mathrm{dt}}=\mathrm{Ra}_{\mathrm{heart}}^{\mathrm{BCAA}}-RDA_{\mathrm{heart}}$ (eq. 3)

BCKA dynamics in heart depends on the balance between BCAA deamination ($\mathrm{RD}A_{\mathrm{heart}}$), BCKA oxidative decarboxylation ($\mathrm{Ro}x_{\mathrm{heart}}$) and BCKA release to the systemic circulation (${Ra}_{heart}^{BCKA}$) rates (eq.4):
$\frac{d\mathrm{BCKA}_{\mathrm{heart}}}{\mathrm{dt}}=\mathrm{Ra}_{\mathrm{heart}}^{\mathrm{BCKA}}+RDA_{\mathrm{heart}}-Rox_{\mathrm{heart}}$ (eq. 4)

It should be stated that the proposed ODE system assumes no impact of cardiac BCAA catabolism on the systemic BCAA or BCKA levels as $\mathrm{Rd}_{\mathrm{heart}}$ is not a component of BCAA ODE; this assumption enables stepwise parameter estimation as described in ‘Methods’ section and is based on relatively low levels of BCKA in the cardiac tissue *vs* systemic circulation.

## Reaction rates

### BCAA consumption and disposal in protein

Placebo-adjusted BCAA and BCKA levels were considered in the model to take into account circadian variation in their plasma levels, resulted from the daily variation in BCAA uptake. Constant BCAA consumption therefore was considered in the model and described as a zero-order process (eq.5).

$Ra_{\mathrm{pl}}=\frac{\mathrm{AM}T_{\mathrm{food}}\cdot BCAA_{\mathrm{perc}}\cdot{10}^{6}}{MW_{\mathrm{BCAA}}\cdot\mathrm{Vd}_{\mathrm{BCAA}}\cdot24\cdot100}$ (eq.5)

Where $\mathrm{AM}T_{\mathrm{food}}$ is a daily food amount, $\mathrm{BCA}A_{\mathrm{perc}}$ – percent of each BCAA in the diet, $MW_{\mathrm{BCAA}}$ – BCAA molecular weight, $\mathrm{Vd}_{\mathrm{BCAA}}$ – volume of BCAA distribution.

BCAA disposal into proteins was set as a first order process which can be expressed from the eq. 1 using steady-state plasma BCAA levels $\mathrm{BCAA}_{\mathrm{pl}}^{\mathrm{stst}}$ and other model parameters (eq. 6):

$\mathrm{RD}A_{\mathrm{pl}}=Ra_{\mathrm{pl}}\cdot\frac{\mathrm{BCAA}_{\mathrm{pl}}}{\mathrm{BCAA}_{\mathrm{pl}}^{\mathrm{stst}}}\cdot\mathrm{prot}_{\mathrm{fr}}$ (eq.6)

Where $\mathrm{BCAA}_{\mathrm{pl}}^{\mathrm{stst}}$ are steady-state levels of individual BCAA, $\mathrm{pro}t_{\mathrm{fr}}$ is a BCAA fraction used for protein synthesis, known from the experimental data (3).

### Cardiac BCAA and BCKA uptake

Reversible influx of BCAA and BCKA from plasma to cardiac tissue was considered in the model. Experimental data indicated rapid equilibration of BCAA and BCKA in plasma and tissues following intravenous BCAA bolus (3) (eq. 7, 8).

$\mathrm{Ra}_{\mathrm{heart}}^{\mathrm{BCAA}}=ktr\cdot(BCAA_{\mathrm{pl}}-BCAA_{\mathrm{heart}})$ (eq.7)

$\mathrm{Ra}_{\mathrm{heart}}^{\mathrm{BCKA}}=ktr\cdot(BCKA_{\mathrm{pl}}-BCKA_{\mathrm{heart}})$ (eq.8)

Where $\mathrm{ktr}$ is a transport rate constant.

### Protein synthesis in cardiac tissue

BCAA expenditure on protein synthesis is considered for the systemic but not for the cardiac compartment for healthy mice based on the data from (3). Reaction of BCAA conversion into the protein was introduced for the TAC state (eq. 9)(4):

$\mathrm{RD}A_{heartpl}={\mathrm{kprot}_{\mathrm{heart}}\cdot BCAA}_{\mathrm{heart}}$ (eq.9)

### BCAA deamination rate

BCAA deamination rates for the individual BCAA in the systemic and cardiac compartments were described using similar equation structures but different parameter values. Enzyme concentrations were assumed to be different between the compartments; biochemical parameters were set for each individual BCAA and BCKA based on the experimental data. Co-substrate levels were assumed to be the same across the compartments due to limited experimental data.

BCAA deamination is a reversible process, catalyzed by BCAT, involving transfer of the BCAA amino group to α-ketoglutarate and formation glutamate (5). A reaction rate equation was derived assuming a ping-pong mechanism (Fig. S1).

$RDA=$ $\mathrm{kcat}_{\mathrm{BCAA}}\cdot BCAT\cdot\frac{(\frac{BCAA\cdot KG}{{\mathrm{Kd}_{\mathrm{BCAA}}\cdot Kd}_{\mathrm{KG}}}-\frac{1}{K\mathrm{eq}_{\mathrm{BCAA}}}\cdot\frac{BCKA\cdot GLU}{{\mathrm{Kd}_{\mathrm{BCKA}}\cdot Kd}_{\mathrm{GLU}}})}{\Delta_{\mathrm{BCAT}}}$ (eq. 10)

where $\mathrm{BCAT}$ is a concentration of BCAT, $\mathrm{BCAA}$ and $\mathrm{BCKA}$ are levels of individual BCAA (Val, Leu, Ile) and BCKA (KIC, KMV, KIV) in the systemic circulation or cardiac tissue. $\mathrm{KG}$ and $\mathrm{GLU}$ are concentrations of KG and GLU, assumed to be constant over time. $\mathrm{kon}_{\mathrm{BCAA}}$, $\mathrm{koff}_{\mathrm{BCAA}}$, $\mathrm{Kd}_{\mathrm{BCAA}}$ are catalytic, equilibrium and dissociation constants for individual BCAA, $\mathrm{Kd}_{\mathrm{BCKA}}$ reflects dissociation constants for BCKA. Denominator $\Delta_{\mathrm{BCAT}}$is represented by eq. 11.

$\Delta_{\mathrm{BCAT}}=1+\frac{\mathrm{KG}}{\mathrm{Kd}_{\mathrm{KG}}}\left( 1+\frac{\mathrm{Leu}}{\mathrm{Kd}_{\mathrm{Leu}}} \right)+\frac{G}{\mathrm{Kd}_{G}}\left( 1+\frac{\mathrm{KIC}}{\mathrm{Kd}_{\mathrm{KIC}}} \right)+\frac{\mathrm{KG}}{\mathrm{Kd}_{\mathrm{KG}}}\left( 1+\frac{\mathrm{Ile}}{\mathrm{Kd}_{\mathrm{Ile}}} \right)+\frac{G}{\mathrm{Kd}_{G}}\left( 1+\frac{\mathrm{KMV}}{\mathrm{Kd}_{\mathrm{KMV}}} \right)+\frac{\mathrm{KG}}{\mathrm{Kd}_{\mathrm{KG}}}\left( 1+\frac{\mathrm{Val}}{\mathrm{Kd}_{\mathrm{Val}}} \right)+\frac{G}{\mathrm{Kd}_{G}}\left( 1+\frac{\mathrm{KIV}}{\mathrm{Kd}_{\mathrm{KIV}}} \right)$ (eq. 11)

Literature data indicate BCAT reaction is near equilibrium (5); to satisfy this observation in the model the following condition, derived from eq. 10, should be fulfilled:

$\frac{\mathrm{BCAA}_{\mathrm{stst}}\cdot KG}{{\mathrm{Kd}_{\mathrm{BCAA}}\cdot Kd}_{\mathrm{KG}}}=\frac{1}{K\mathrm{eq}_{\mathrm{BCAA}}}\cdot\frac{\mathrm{BCKA}_{\mathrm{stst}}\cdot GLU}{{\mathrm{Kd}_{\mathrm{BCKA}}\cdot Kd}_{\mathrm{GLU}}}$ (eq. 12)

From this equation level of the co-substrate (KG or GLU) can be expressed:

$GLU=\frac{\mathrm{BCAA}_{\mathrm{stst}}\cdot KG\cdot K\mathrm{eq}_{\mathrm{BCAA}}{{\cdot\mathrm{Kd}}_{\mathrm{BCKA}}\cdot Kd}_{\mathrm{GLU}}}{{\mathrm{Kd}_{\mathrm{BCAA}}\cdot Kd}_{\mathrm{KG}}{\cdot BCKA}_{\mathrm{stst}}}$ (eq. 13)

Leu and KIC steady-state levels were used to derive glutamate concentration.

### BCKA oxidation rate

BCKA oxidation rate equation is similar across the organs and individual BCKA and is represented by irreversible rate (eq. 14):

$Rox=\frac{k_{\mathrm{BCKA}}\cdot BCKD\cdot BCKA}{(1+\frac{\mathrm{KIC}}{\mathrm{Kd}_{\mathrm{KIC}}^{\mathrm{BCKD}}}+\frac{\mathrm{KMV}}{\mathrm{Kd}_{\mathrm{KMV}}^{\mathrm{BCKD}}}+\frac{\mathrm{KIV}}{\mathrm{Kd}_{\mathrm{KIV}}^{\mathrm{BCKD}}}){\cdot Kd}_{\mathrm{BCKA}}^{\mathrm{BCKD}}}$ (eq. 14)

Where $k_{\mathrm{BCKA}}$ and $\mathrm{Kd}_{\mathrm{BCKA}}^{\mathrm{BCKD}}$ are catalytic and dissociation constants, different across individual BCKA.

### BCKD phosphorylation and dephosphorylation rate

Balance between active (dephosphorylated) and inactive (phosphorylated) BCKD forms depends on the activities of BCKD kinase (BCKDK) and protein phosphatase 2Cm (PP2Cm) and can be characterized using ODE eq. 15 and 16:

$\dot{\frac{\mathrm{dBCKD}}{\mathrm{dt}}}=-V_{\mathrm{BCKDK}}+V_{PP2Cm}$ (eq. 15)

$\dot{\frac{\mathrm{dpBCKD}}{\mathrm{dt}}}=V_{\mathrm{BCKDK}}-V_{PP2Cm}$ (eq. 16)

BCKD phosphorylation is a bisubstrate reaction, which can be inhibited by BCKA (mainly, KIC) (6) as well as pharmacological agents such as BT2 (Fig. S2 eq. 17):

$V_{\mathrm{BCKDK}}=k_{\mathrm{BCKDK}}\cdot BCKDK \cdot\frac{\mathrm{BCKD}\cdot\mathrm{ATP}}{{{Kd}_{\mathrm{BCKD}}\cdot Kd}_{\mathrm{ATP}}\cdot\boldsymbol{\Delta}}$ (eq.17)

Where $\Delta=1+\frac{\mathrm{ATP}}{{Kd}_{\mathrm{ATP}}}\cdot\left( 1+\frac{\mathrm{BCKD}}{{Kd}_{\mathrm{BCKD}}}+\frac{\mathrm{KIC}}{{Kd}_{\mathrm{KIC}}}+\frac{BT2}{{Kd}_{BT2}} \right)+\frac{\mathrm{BCKD}}{{Kd}_{\mathrm{BCKD}}}\cdot\left( 1+\frac{K\mathrm{IC}}{{Kd}_{\mathrm{KIC}}}+\frac{BT2}{{Kd}_{BT2}} \right)+\frac{K\mathrm{IC}}{{Kd}_{K\mathrm{IC}}}+\frac{BT2}{{Kd}_{BT2}}$

BCKDK dephosphorylation is described using eq. 18:

$V_{PP2Cm}=k_{PP2Cm}\cdot PP2Cm\cdot\frac{\mathrm{pBCKD}}{{Kd}_{PP2Cm}\mathbf{+}\mathrm{pBCKD}}$ (eq.18)

Assuming fast equilibrium between phosphorylated and active BCKD form in case of no KIC and BT2 inhibition we can express BCKD level via other parameters (eq. 19):

$k_{\mathrm{BCKDK}}\cdot BCKDK \cdot\frac{\mathrm{BCKD}\cdot\mathrm{ATP}}{{{Kd}_{\mathrm{BCKD}}\cdot Kd}_{\mathrm{ATP}}\cdot\boldsymbol{\Delta}}\mathbf{=}k_{PP2Cm}\cdot PP2Cm\cdot\frac{\mathrm{pBCKD}}{{Kd}_{PP2Cm}\mathbf{+}\mathrm{pBCKD}}$ (eq.19)

Let’s introduce following notations:

$a=\frac{\mathrm{ATP}}{{{Kd}_{\mathrm{BCKD}}\cdot Kd}_{\mathrm{ATP}}}$

$b=\frac{1}{{Kd}_{\mathrm{BCKD}}}\left( \frac{\mathrm{ATP}}{{Kd}_{\mathrm{ATP}}}+\left( 1+\frac{K\mathrm{IC}}{{Kd}_{\mathrm{KIC}}}+\frac{BT2}{{Kd}_{BT2}} \right) \right)$

$c=\left( 1+\frac{\mathrm{ATP}}{{Kd}_{\mathrm{ATP}}} \right)\left( 1+\frac{\mathrm{KIC}}{{Kd}_{\mathrm{KIC}}}+\frac{BT2}{{Kd}_{BT2}} \right)$

${r=k}_{\mathrm{BCKD}}\cdot BCKDK/{(k}_{PP2Cm}\cdot PP2Cm$)

$$\mathrm{BCKD}_{\mathrm{tot}}=BCKD+pBCKD$$

The eq. 19 then can be re-written (eq. 20):

$r\cdot\frac{\mathrm{BCKD}\cdot a}{(b\cdot BCKD+c)}\mathbf{=}\frac{(\mathrm{BCKD}_{\mathrm{tot}}-BCKD)}{{Kd}_{PP2Cm}\mathbf{+}(\mathrm{BCKD}_{\mathrm{tot}}-BCKD)}$ (eq.20)

Eq. 19 can be re-written as a quadratic equation (eq. 21):

$\left( -a\cdot r+b \right)\cdot\mathrm{BCKD}^{2}+\left( a\cdot r\cdot({Kd}_{PP2Cm}+\mathrm{BCKD}_{\mathrm{tot}})-\mathrm{BCKD}_{\mathrm{tot}}\cdot b+c \right)\cdot BCKD-\mathrm{BCKD}_{\mathrm{tot}}\cdot c=0$

(eq.21)

Active BCKD level can be expressed from the eq. 21:

$BCKD=\frac{-k2+\sqrt{{k2}^{2}-4\cdot k1\cdot k3}}{2\cdot k1}$ (eq. 22)

Where $k1=\left( -a\cdot r+b \right)$, $k2=\left( a\cdot r\cdot({Kd}_{PP2Cm}+\mathrm{BCKD}_{\mathrm{tot}})-\mathrm{BCKD}_{\mathrm{tot}}\cdot b+c \right)$ and ${k3=BCKD}_{\mathrm{tot}}\cdot c$.

### Cardiac BCAA effect on LVEF

A simple disease progression model was used to characterize LVEF dynamics following TAC surgery. LVEF change from baseline $\mathrm{CF}B_{\mathrm{tr}}$ was described using time-dependent function (eq. 23):

$\mathrm{CF}B_{\mathrm{tr}}=-kpr\cdot t-\mathrm{LVE}F_{\mathrm{dec}}$ (eq. 23)

Where $\mathrm{kpr}$ is a parameter, characterizing BCAA-independent LVEF decrease in time, $\mathrm{LVE}F_{\mathrm{dec}}$ represents BCAA-mediated LVEF decrease (eq. 24):

$\frac{\mathrm{dLVE}F_{\mathrm{dec}}}{\mathrm{dt}}=lvef_{\mathrm{to}}\cdot(\frac{BCAA-BCAA_{\mathrm{bln}}}{\mathrm{BCA}A_{\mathrm{bln}}}\cdot keff-LVEF_{\mathrm{dec}})$ (eq. 24)

Where $\mathrm{keff}$ and $\mathrm{lve}f_{\mathrm{to}}$ are parameters, characterizing magnitude and delay of BCAA impact on LVEF, respectively.

### BT2 pharmacokinetic model

A simple one-compartment model with linear absorption and elimination rates was set up (eq. 25, 26):

$\frac{\mathrm{dBT}2_{\mathrm{adm}}}{\mathrm{dt}}=-ka\cdot BT2_{\mathrm{adm}}$ (eq. 25)

$\frac{\mathrm{dBT}2_{\mathrm{pl}}}{\mathrm{dt}}=ka\cdot BT2_{\mathrm{adm}}-kel\cdot BT2_{\mathrm{pl}}$ (eq. 26)

Plasma BT2 concentration was calculated using eq. 27:

$BT2=BT2_{\mathrm{pl}}/(Vd_{BT2}\cdot MW_{BT2})\cdot{10}^{6}$ (eq. 27)

# Supplementary figures


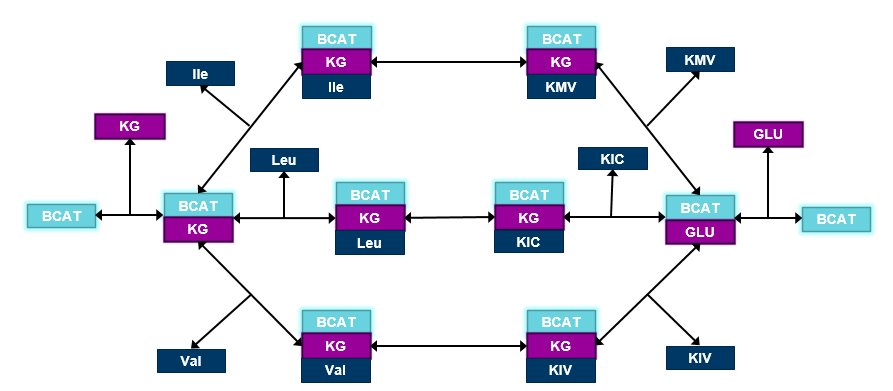


**Figure S1.** Reversible BCAA deamination reaction schematics.


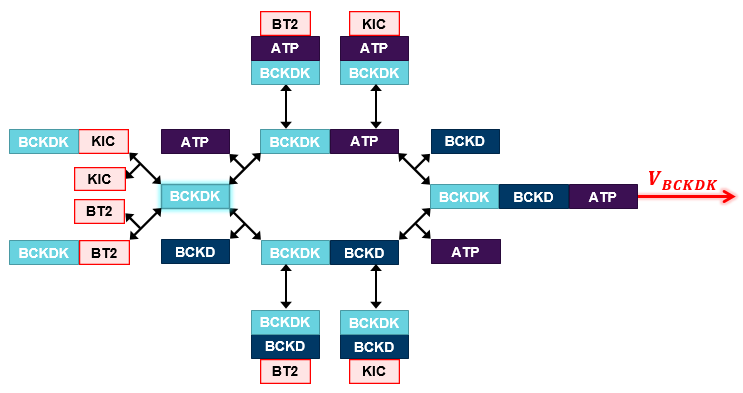


**Figure S2.** BCKD activity regulation by different enzymes.


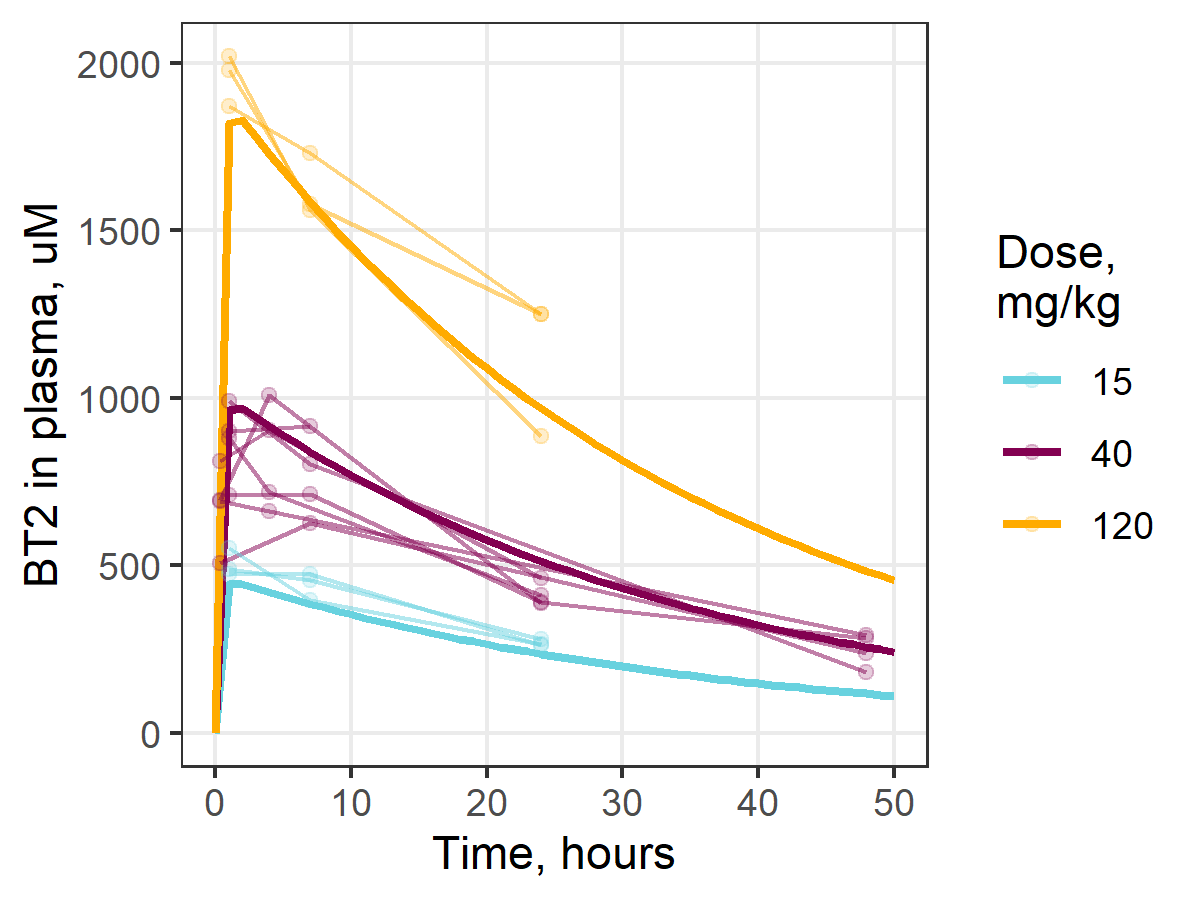


**Figure S3.** BT2 PK data reproduction. Data from individual animals are shown by thin lines, model-based simulations are represented by thick lines.


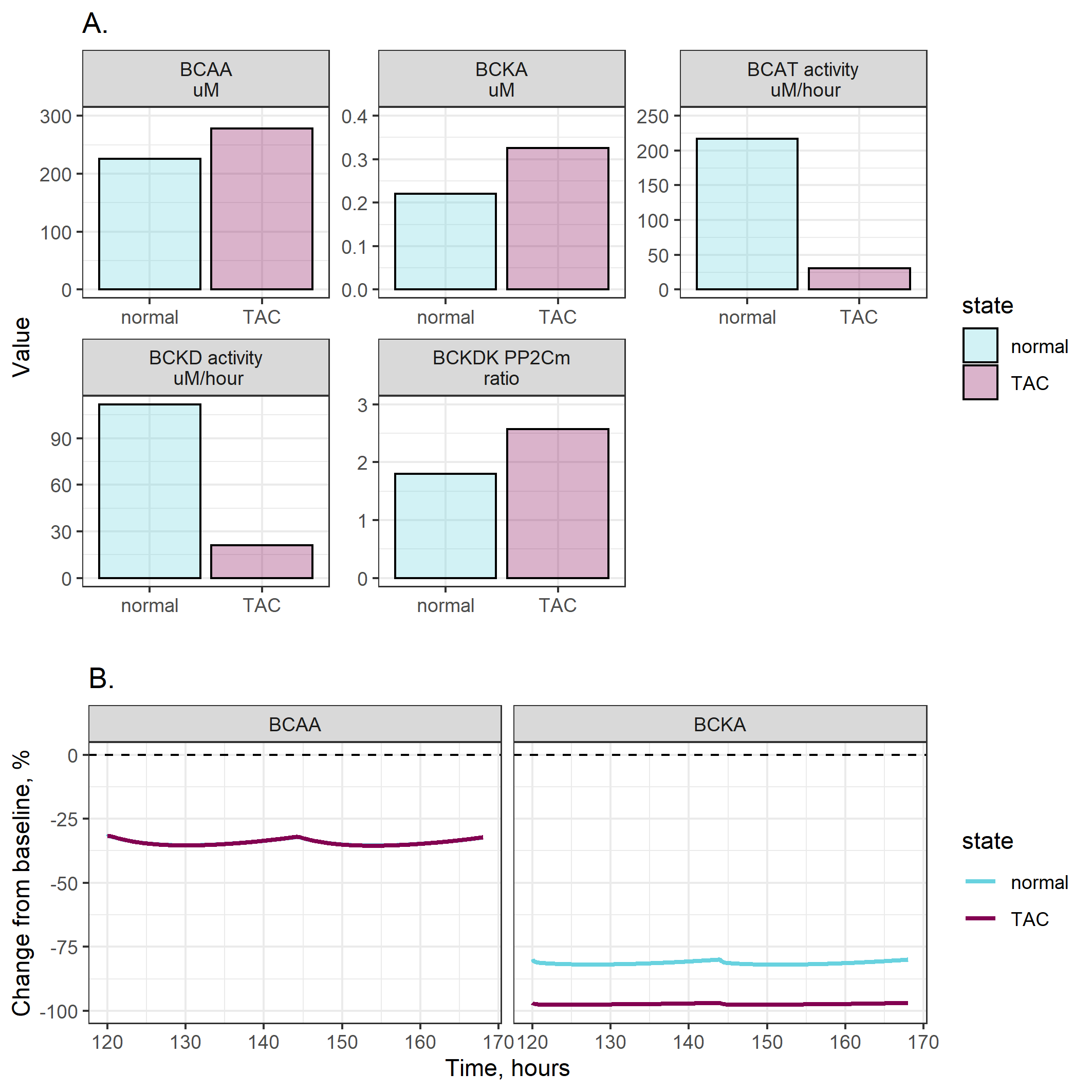
**Figure S4.** Comparison of BCAA catabolism in normal and TAC states in the model. BCAT and PP2Cm activities were set 14% and 30% from normal based on (4), protein synthesis from BCAA in cardiac tissue was introduced. (A) Steady-state metabolite levels and enzyme activities in cardiac tissue. (B) BT2 effect on cardiac BCAA and BCKA levels

**
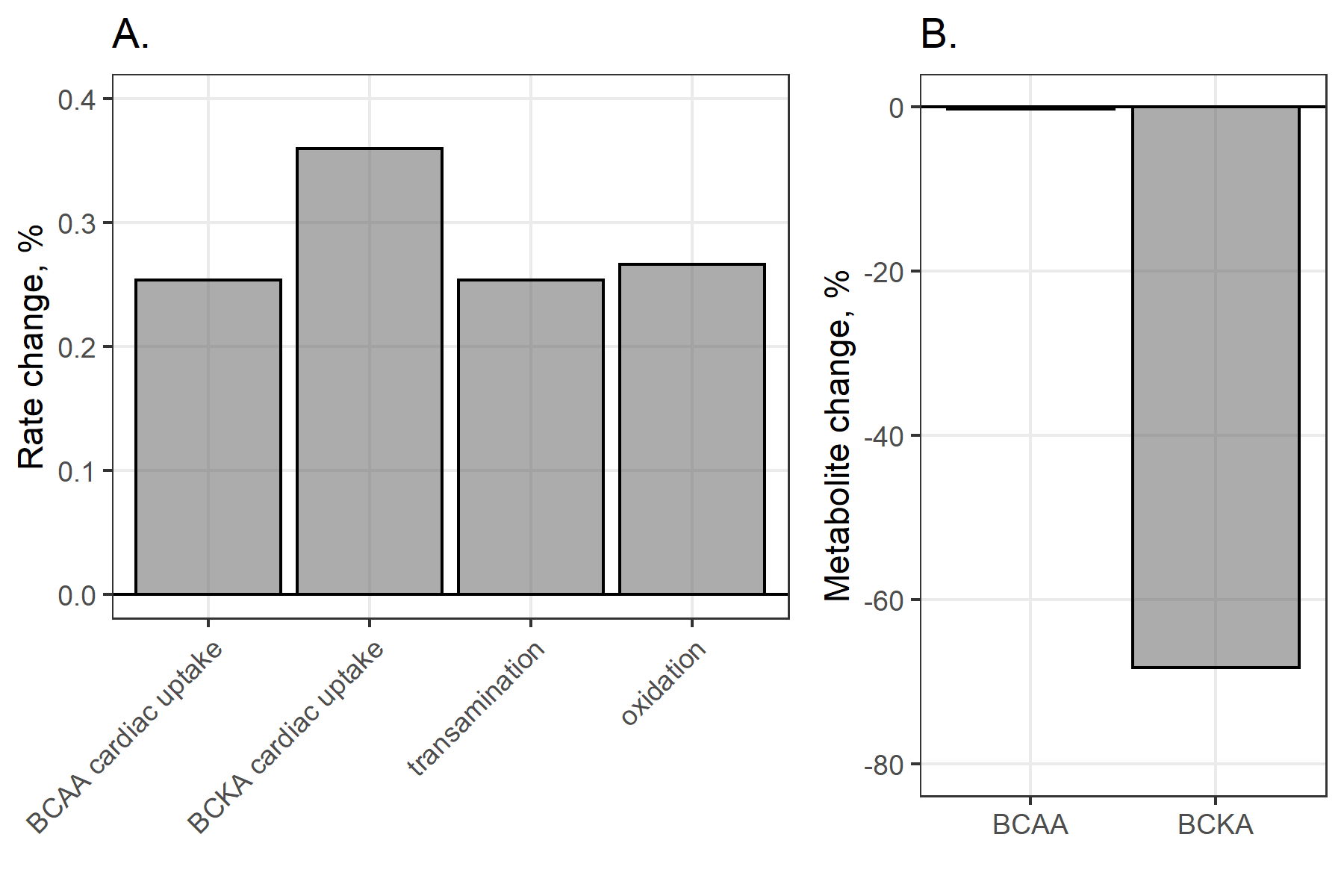
**

**Figure S5.** BT2 treatment impact on cardiac biomarkers in case when systemic BT2 effects are nullified. **A.** Change in cardiac BCAA and BCKA catabolic rates; **B.** Change in cardiac BCAA and BCKA levels.

# Supplementary tables

**Table S1.** Summary of the experimental data

| **Experiment** | **Dosing regimen** | **Measurements** | **Sampling** |
| --- | --- | --- | --- |
| **BS001805-57** | 40 mg/kg at time 0 and 24 hours | BT2, BCAA, BCKA in plasma (healthy mice) | 0, 4, 24, 48 hours |
| **BS001805-66** | 40 mg/kg at time 0 hours |  | 0, 20 min, 1, 4, 7, 24, 48 hours |
| **BS001805-67** | 15 and 120 mg/kg at time 0 hours |  | 0, 1, 7, 24 hours |
| **BS001805-57** | Vehicle | BCAA and BCKA in cardiac tissue (healthy mice) | 0 hours |
| **(4)** | Vehicle | BCAA and BCKA in cardiac tissue (TAC mice) | 0 hours |
| **(2)** | 0 or 40 mg/kg starting 2 weeks after TAC, once daily for 6 weeks | LVEF dynamics (TAC mice) | At the end of the experiment (6 weeks of BT2 dosing) |

**Table S2.** Parameter values

| Parameter | Unit | Description | Value (RSE)* | Estimation | Source |
| --- | --- | --- | --- | --- | --- |
| **Biochemical parameters (BCAA deamination)** | | | | | |
| $k_{\mathrm{Leu}}$ | 1/s | Catalytic constant | 337 | Fixed | (7) |
| $k_{\mathrm{Ile}}$ | 1/s | Catalytic constant | 371 | Fixed |  |
| $k_{\mathrm{Val}}$ | 1/s | Catalytic constant | 290 | Fixed |  |
| $\mathrm{Kd}_{\mathrm{Leu}}$ | uM | Dissociation constant | 1600 | Fixed |  |
| $\mathrm{Kd}_{\mathrm{Ile}}$ | uM | Dissociation constant | 1300 | Fixed |  |
| $\mathrm{Kd}_{\mathrm{Val}}$ | uM | Dissociation constant | 1600 | Assumed same as for Leu |  |
| $\mathrm{Kd}_{\mathrm{KIC}}^{\mathrm{BCAT}}$ | uM | Dissociation constant | 300 | Fixed |  |
| $\mathrm{Kd}_{\mathrm{KMV}}^{\mathrm{BCAT}}$ | uM | Dissociation constant | 200 | Fixed |  |
| $\mathrm{Kd}_{\mathrm{KIV}}^{\mathrm{BCAT}}$ | uM | Dissociation constant | 300 | Assumed same as for KIC |  |
| $\mathrm{Kd}_{\mathrm{KG}}$ | uM | Dissociation constant | 8300 | Fixed |  |
| $\mathrm{Kd}_{\mathrm{Glu}}$ | uM | Dissociation constant | 22700 | Fixed |  |
| $\mathrm{Keq}_{\mathrm{Leu}}$ | uM | Equilibrium constant | 1500 | Fixed |  |
| $\mathrm{Keq}_{\mathrm{Ile}}$ | uM | Equilibrium constant | 1020 | Fixed |  |
| $\mathrm{Keq}_{\mathrm{Val}}$ | uM | Equilibrium constant | 1500 | Fixed |  |
| **Biochemical parameters (BCKA oxidative decarboxylation)** | | | | | |
| $k_{\mathrm{KIC}}$ | 1/s | Catalytic constant | 3.3 | Fixed | (8) |
| $k_{\mathrm{KMV}}$ | 1/s | Catalytic constant | 3.3 |  |  |
| $k_{\mathrm{KIV}}$ | 1/s | Catalytic constant | 5.5 |  |  |
| $\mathrm{Kd}_{\mathrm{KIC}}^{\mathrm{BCKD}}$ | uM | Dissociation constant | 50 |  |  |
| $\mathrm{Kd}_{\mathrm{KMV}}^{\mathrm{BCKD}}$ | uM | Dissociation constant | 37 |  |  |
| $\mathrm{Kd}_{\mathrm{KIV}}^{\mathrm{BCKD}}$ | uM | Dissociation constant | 55 |  |  |
| **Biochemical parameters (BCKD phosphorylation/dephosphorylation)** | | | | | |
| $Kd_{\mathrm{ATP}}$ | uM | Dissociation constant | 33 | Fixed | (6) |
| $Kd_{\mathrm{BCKD}}$ | uM | Dissociation constant | 10 |  |  |
| $Ki_{\mathrm{KIC}}$ | uM | Inhibition constant BCKDK by KIC | 14 |  |  |
| $Kd_{PP2Cm}$ | uM | Dissociation constant | 57.8 | Fixed | (9) |
| **Diet-related parameters** | | | | | |
| $\mathrm{AM}T_{\mathrm{food}}$ | g/day | Daily food consumption | 5 | - | - |
| $\mathrm{Leu}_{\mathrm{perc}}$ | % | Leu percentage in food | 0.9 | Fixed (R70 Growth and maintenance feed for rat and mouse) | (12) |
| $\mathrm{Ile}_{\mathrm{perc}}$ | % | Ile percentage in food | 0.6 |  |  |
| $\mathrm{Val}_{\mathrm{perc}}$ | % | Val percentage in food | 0.6 |  |  |
| **Organism specific parameters (systemic)** | | | | | |
| $\mathrm{pro}t_{\mathrm{fr}}$ | - | BCAA fraction, disposed in protein | 0.5 | Assumed based on the experimental data | (3) |
| $\mathrm{Leu}_{\mathrm{pl}}^{\mathrm{stst}}$ | uM | Steady-state level | 129.6 | Mean observed baseline values | Table S1 |
| $\mathrm{Ile}_{\mathrm{pl}}^{\mathrm{stst}}$ | uM | Steady-state level | 86.9 |  |  |
| $\mathrm{Val}_{\mathrm{pl}}^{\mathrm{stst}}$ | uM | Steady-state level | 215.8 |  |  |
| $\mathrm{KIC}_{\mathrm{pl}}^{\mathrm{stst}}$ | uM | Steady-state level | 19.0 |  |  |
| $\mathrm{KMV}_{\mathrm{pl}}^{\mathrm{stst}}$ | uM | Steady-state level | 21.2 |  |  |
| $\mathrm{KIV}_{\mathrm{pl}}^{\mathrm{stst}}$ | uM | Steady-state level | 16.9 |  |  |
| $\mathrm{KG}$ | uM | Co-substrate level | 2.158 | Calculated | (10) |
| $\mathrm{ATP}$ | uM | Co-substrate level | 32.37 |  |  |
| $\mathrm{Vd}_{\mathrm{BCAA}}$ | L | BCAA volume of distribution | 0.675 (19.2) | Estimated based on the data | Table S1 |
| $\mathrm{BCAT}$ | uM | BCAT level | 0.765 (18.3) |  |  |
| $\mathrm{BCKDtot}$ | uM | Total BCKD level | 20 (fixed) |  |  |
| $R$ | - | BCKDK to PP2Cm activity ratio | 4590 (18.7) |  |  |
| **Organism-specific parameters (heart, healthy mice)** | | | | | |
| $\mathrm{Vheart}$ | L | Cardiac tissue volume | 9e-5 | Fixed | PK-Sim® software |
| $\mathrm{ktr}$ | 1/hour | BCAA and BCKA plasma to heart rate constant | 21 | Assumed based BCAA and BCKA dynamics in plasma and tissues following intravenous BCAA bolus | (3) |
| $\mathrm{BCA}T_{h}$ | uM | BCAT level | 217 (0.0425) | Estimated based on cardiac BCAA and BCKA levels and BCAA percentage, being oxidized in the heart | (3) |
| $\mathrm{BCKDto}t_{h}$ | uM | Total BCKD level | 367 (2.52) |  |  |
| $r_{h}$ | - | BCKDK to PP2Cm activity ratio | 1.8 | Fixed to have 30% BCKD active fraction | (3,11) |
| $\mathrm{Kpr}$ | 1/hour | TAC-induced LVEF decline rate constant | 0.010375 (12.9) | Estimated based on the data | (2) |
| $\mathrm{keff}$ | - | BCAA effect on LVEF decline | 31.3 (13.8) |  |  |
| $\mathrm{lve}f_{\mathrm{to}}$ | 1/hour | Delay in BCAA effect on LVEF decline | 0.00432 (29.7) |  |  |
| $\mathrm{kprot}_{\mathrm{heart}}$ | 1/hour | BCAA conversion into protein | 0 | Set based on the data | (3) |
| **Organism-specific parameters (heart, TAC mice)** | | | | | |
| $\mathrm{BCA}T_{h}$ | uM | BCAT level | 30.37 | Set based on the data | (4) |
| $r_{h}$ | - | BCKDK to PP2Cm activity ratio | 2.57 | Set based on the data |  |
| $\mathrm{kprot}_{\mathrm{heart}}$ | 1/hour | BCAA conversion into protein | 15 | Set to have 50% increase in cardiac BCKA |  |
| **BT2-specific parameters** | | | | | |
| $\mathrm{Ka}$ | 1/hour | Absorption constant | 3.4 (17.5) | Estimated based on BT2 PK data | - |
| $ED50$ | Mg | Drug dose, associated with 50% bioavailability | 95.8 (16.3) |  |  |
| $\mathrm{CL}$ | ml/hour/kg | Clearance | 3.24 (6.95) |  |  |
| $\mathrm{Vd}$ | ml/kg | Volume of distribution | 112 (6.61) |  |  |
| $\mathrm{MW}$ | g/mol | Molecular weight | 247.1 | Fixed | (12) |
| $Kd_{BT2}$ | nM | BCKDK inhibition by BT2: dissociation constant | 406 (6.57) | Estimated based on the data | - |
| $\mathrm{Nh}_{BT2}$ | - | BCKDK inhibition by BT2: Hill coefficient | 2.11 (8.96) |  |  |

# References

1. Olson KC, Chen G, Lynch CJ. Quantification of branched-chain keto acids in tissue by ultra fast liquid chromatography–mass spectrometry. Anal Biochem. 2013 Aug;439(2):116–22.

2. Chen M, Gao C, Yu J, Ren S, Wang M, Wynn RM, et al. Therapeutic Effect of Targeting Branched‐Chain Amino Acid Catabolic Flux in Pressure‐Overload Induced Heart Failure. J Am Heart Assoc [Internet]. 2019 Jun 4 [cited 2020 Nov 16];8(11). Available from: https://www.ahajournals.org/doi/10.1161/JAHA.118.011625

3. Neinast MD, Jang C, Hui S, Murashige DS, Chu Q, Morscher RJ, et al. Quantitative Analysis of the Whole-Body Metabolic Fate of Branched-Chain Amino Acids. Cell Metab. 2019 Feb 5;29(2):417-429.e4.

4. Sun H, Olson KC, Gao C, Prosdocimo DA, Zhou M, Wang Z, et al. Catabolic Defect of Branched-Chain Amino Acids Promotes Heart Failure. Circulation. 2016 May 24;133(21):2038–49.

5. Holeček M. Branched-chain amino acids in health and disease: metabolism, alterations in blood plasma, and as supplements. Nutr Metab. 2018 May 3;15(1):33.

6. Paxton R, Harris RA. Regulation of branched-chain alpha-ketoacid dehydrogenase kinase. Arch Biochem Biophys. 1984 May 15;231(1):48–57.

7. Yennawar NH, Islam MM, Conway M, Wallin R, Hutson SM. Human mitochondrial branched chain aminotransferase isozyme: structural role of the CXXC center in catalysis. J Biol Chem. 2006 Dec 22;281(51):39660–71.

8. Pettit FH, Yeaman SJ, Reed LJ. Purification and characterization of branched chain alpha-keto acid dehydrogenase complex of bovine kidney. Proc Natl Acad Sci U S A. 1978 Oct;75(10):4881–5.

9. Wynn RM, Li J, Brautigam CA, Chuang JL, Chuang DT. Structural and biochemical characterization of human mitochondrial branched-chain α-ketoacid dehydrogenase phosphatase. J Biol Chem. 2012 Mar 16;287(12):9178–92.

10. Ogawa T, Washio J, Takahashi T, Echigo S, Takahashi N. Glucose and glutamine metabolism in oral squamous cell carcinoma: insight from a quantitative metabolomic approach. Oral Surg Oral Med Oral Pathol Oral Radiol. 2014 Aug;118(2):218–25.

11. Suryawan A, Hawes JW, Harris RA, Shimomura Y, Jenkins AE, Hutson SM. A molecular model of human branched-chain amino acid metabolism. Am J Clin Nutr. 1998 Jul 1;68(1):72–81.

12. Tso SC, Gui WJ, Wu CY, Chuang JL, Qi X, Skvora KJ, et al. Benzothiophene carboxylate derivatives as novel allosteric inhibitors of branched-chain α-ketoacid dehydrogenase kinase. J Biol Chem. 2014 Jul 25;289(30):20583–93.
